# JAK3-STAT pathway blocking benefits in experimental lupus nephritis

**DOI:** 10.1186/s13075-016-1034-x

**Published:** 2016-06-08

**Authors:** Èlia Ripoll, Laura de Ramon, Juliana Draibe Bordignon, Ana Merino, Nuria Bolaños, Montse Goma, Josep M. Cruzado, Josep M. Grinyó, Juan Torras

**Affiliations:** Laboratori 4120. Nefrologia Experimental, 4a Planta Pavelló Govern, Universitat de Barcelona. Campus Bellvitge, Institut d’Investigació Biomèdica de Bellvitge (IDIBELL). Departament de Nefrologia, Hospital Universitari de Bellvitge, E-08907 L’Hospitalet, Barcelona, Spain; Departament d’Anatomia Patològica, Hospital Universitari de Bellvitge, E-08907 L’Hospitalet, Barcelona, Spain

**Keywords:** Autoimmunity, Glomerulonephritis, JAK3 inhibitor, Kidney infiltrate, Lupus nephritis

## Abstract

**Background:**

Lupus nephritis (LN) is a complex chronic autoimmune disease of unknown etiology characterized by loss of tolerance against several self-antigens. Cytokines are known to be central players in LN pathogenesis. The Janus kinase-signal transducer and activator of transcription (JAK-STAT) pathway is one important pathway that mediates signal transduction of several cytokines. In this study, we examined the pathogenic role of this pathway and how CP-690,550 treatment influences LN outcome.

**Methods:**

Six-month-old NZB/NZWF1 mice were divided into two different treatment groups: (1) control animals given vehicle treatment, cyclophosphamide, and mycophenolate mofetil treatment as positive controls of the therapy and (2) mice treated with CP-690,550, a JAK3 inhibitor. Mice were treated for 12 weeks. We evaluated renal function, anti-double-stranded DNA (anti-dsDNA) antibody, renal histology changes, kidney complement and immunoglobulin G (IgG) deposits, T-cell and macrophage infiltration, kidney inflammatory gene expression, and circulating cytokine changes.

**Results:**

CP-690,550 treatment significantly reduced proteinuria and improved renal function and histological lesions of the kidney. Compared with vehicle-treated animals, those undergoing CP-690,550 treatment showed significantly diminished anti-dsDNA antibody and complement component C3 and IgG deposition in glomeruli. We also observed a significant reduction of T-cell and macrophage infiltration. Kidney gene expression revealed a reduction in inflammatory cytokines and complement and related macrophage-attracting genes. Circulating inflammatory cytokines were also reduced with treatment.

**Conclusions:**

On the basis of our results, we conclude that the JAK-STAT pathway is implicated in the progression of renal inflammation in NZB/WF1 mice and that targeting JAK3 with CP-690,550 is effective in slowing down the course of experimental LN. Thus, CP-690,550 could become a new therapeutic tool in LN and other autoimmune diseases.

## Background

Systemic lupus erythematosus (SLE) is a chronic systemic autoimmune disorder characterized by loss of tolerance against nuclear autoantigens, anti-double-stranded DNA (anti-dsDNA) antibody production, immune complex (IC) deposition, and leukocyte infiltration in many target organs, as well as activated B and T cells [1, 2]. Lupus nephritis (LN) is an IC glomerulonephritis categorized as one of the most serious complications of SLE and is also one of the strongest predictors of a poor prognosis. LN can lead to severe proteinuria, hypertension, chronic renal failure, and, finally, end-stage kidney disease.

It is well known that the cytokine milieu is integrally involved in the pathogenesis of autoimmune diseases [3, 4]. Multiple cytokines, such as interferons (IFNs), tumor necrosis factor (TNF)-α interleukin (IL)-6, IL-12, IL-10, and IL-17, are implicated in the initiation, progression, and development of LN. Serum levels of several of them have been found to be elevated in patients with active SLE and in murine LN models [5–8], and abnormally high levels are associated with more severe disease and severity [9, 10]. IL-6 is a proinflammatory cytokine that plays a central regulatory role in the development, maturation, survival, and immunoglobulin secretion of long-lived plasma cells [11]. IL-17 amplifies the immune response by inducing the local production of chemokines and cytokines, recruiting neutrophils and monocytes, augmenting the production of autoantibodies, and aggravating the inflammation and damage of target organs [12].

One of the best-understood mechanisms by which cytokines activate signals, thus eliciting specific responses in target cells, involves enzymes such as Janus kinases (JAKs) and transcriptional factors such as signal transducers and activators of transcription (STATs). The JAK-STAT signaling pathways are used by all type I and type II cytokine receptors. Following ligation, JAK phosphorylates the cytoplasmic tail of its receptor, which becomes activated, leading to the recruitment of STATs. Afterward, STATs translocate into the nucleus, where they regulate the expression of numerous genes [13]. The pathological consequences of deregulated JAK/STAT signaling have been widely documented in many animal models of chronic inflammatory and autoimmune diseases as well as cancers [14]. Some polymorphisms of JAK/STAT genes have also been associated with susceptibility to SLE [15, 16]. This association with severe immune dysfunction reveals the key role of this pathway in the induction and regulation of immune responses.

The JAK3 inhibitor CP-690,550 is a drug currently approved for the treatment of rheumatoid arthritis. It is currently also being evaluated for the treatment of other autoimmune diseases, as well as for the prevention of organ transplant rejection [17, 18]. Very few studies have assessed this family of drugs in the context of LN. CP-690,550 is an orally available compound that specifically binds to the adenosine triphosphate (ATP)-binding pocket of JAK3, though recent studies have shown that CP-690,550 is also able to bind to the ATP pocket in both JAK1 and JAK2 [19, 20]. Therefore, CP-690,550 is currently categorized as a pan-JAK inhibitor preferentially inhibiting JAK3 and JAK1, and to a lesser extent JAK2.

In contrast to the relatively ubiquitous expression of JAK1, JAK2, and Tyk2, JAK3 has a more restricted and regulated expression. JAK3 is selectively expressed in hematopoietic cells, natural killer cells, thymocytes, T and B cells, and myeloid cells, and mutation in this kinase results in a loss of immunological function and in severe combined immunodeficiency [13]. We hypothesized that blocking the enzymatic activity of JAK3 might also result in immunosuppression, thereby offering a novel, attractive, and promising strategy in the treatment of inflammatory immune-mediated diseases. Therefore, in the present study, we examined the effects of blocking the JAK-STAT pathway on disease progression and renal lesions in NZB/WF1 mice via in vivo administration of CP-690,550. We further evaluated whether this blockade interferes only locally in the progression of LN or also in downstream mechanisms of SLE development.

## Methods

### Mice, study design, and follow-up

Five-month-old NZB/WF1 mice (JAX®; Charles River Laboratories, Barcelona, Spain) were randomly assigned to four groups. At 6 months of age, treatment was initiated as follows, by group: intraperitoneal cyclophosphamide (CYP) 50 mg/kg/10 days (*n* = 14), subcutaneous CP-690,550 (CP, XELJANZ® [tofacitinib]; Pfizer, New York, NY, USA) 48 mg/kg/day (*n* = 8), oral mycophenolate mofetil (MMF, CellCept®; ROCHE FARMA, Madrid, Spain) 30 mg/kg/daily (*n* = 8), and subcutaneous PBS treatment (*n* = 14) as a control group. Mice were treated for 12 weeks. Their body weight was determined twice monthly from the beginning to the end of follow-up. Mice were placed in metabolic cages to collect 24-h urine specimens before the onset of treatment, and monthly thereafter. Blood was obtained from the tail vein at monthly intervals. Kidneys were processed for histological and biochemical studies at the end of the study or at death.

The experiments were carried out in accordance with current European Union legislation on animal experimentation and were approved by the animal experimentation ethics committee, the University of Barcelona Institutional Ethics Committee for Animal Research, and the Animal Experimentation Commission of the Generalitat de Catalunya (Catalonian government). The mice were housed in a room at constant temperature with a 12-h dark/12-h light cycle, were given free access to water, and were fed a standard laboratory diet.

### Proteinuria, albuminuria, and renal function

Twenty-four-hour urinary protein was determined by Pyrogallol red reaction (AU400 Clinical Chemistry System; Beckman Coulter, Brea, CA, USA) in the Veterinary Clinical Biochemistry Laboratory of Universitat Autonoma de Barcelona. Twenty-four-hour urinary albumin was determined using a commercially available enzyme-linked immunosorbent assay (ELISA) kit (Active Motif, Carlsbad, CA, USA) according to the manufacturer’s instructions.

### RNA extraction, reverse transcription, and gene expression analysis using quantitative real-time polymerase chain reactions

For molecular studies, the kidney was immediately snap-frozen in liquid nitrogen and stored at −80 °C. RNA was extracted from kidneys using the PureLink™ RNA Mini Kit (Ambion/Thermo Fisher Scientific, Barcelona, Spain) according to the manufacturer’s instructions. RNA purity was analyzed using a NanoDrop ND-1000 V3.3 spectrophotometer (NanoDrop Technologies, Wilmington, DE, USA). RNA was stored at −80 °C. A total of 500 ng of RNA was used for reverse transcription with a high-capacity cDNA reverse transcription kit (Applied Biosystems, Warrington, UK) in accordance with the manufacturer’s instructions. Tissue expression levels of the following mediators of immunity and inflammation were quantified by TaqMan real-time polymerase chain reaction (ABI Prism® 7700, Applied Biosystems, Spain) using the comparative C_t_ method: complement component 3 (C3), chemokine (C-C motif) ligand 2 (CCL2), CCL5, CD40L, IL-2, IL-6, Toll-like receptor 9 (TLR9), vascular cell adhesion molecule (VCAM)-1, STAT1, STAT2, STAT3, STAT4, and STAT5a.

### Plasma ELISA for anti-DNA antibodies and serum cytokine analysis

Levels of anti-DNA antibodies were measured using a commercially available ELISA kit (Alpha Diagnostic International, San Antonio, TX, USA) according to the manufacturer’s instructions. Serum IL-12, TNF-α, IFN-γ, and monocyte chemoattractant protein (MCP)-1 cytokines were measured using the BD FACSCanto flow cytometer with a cytometric bead array kit (BD CBA mouse inflammation kit) according to the manufacturer’s instructions (BD Biosciences, San Jose, CA, USA). Data were acquired and analyzed using BD FCAP software and CBA software (BD Biosciences). Serum IL-17 (R&D Systems, Minneapolis, MN, USA) and IFN-α (PBL Assay Science, Piscataway, NJ, USA) cytokine levels were quantified using commercially available ELISA kits according to the manufacturers’ instructions.

### Renal lupus histopathology

For histological analyses, 1- to 2-mm-thick coronal slices of kidney tissue were fixed in 4 % formaldehyde and embedded in paraffin. For light microscopy, 3- to 4-μm-thick tissue sections were stained with hematoxylin and eosin stain and periodic acid-Schiff stain. To determine the extent of renal damage, two blinded pathologists analyzed all kidney biopsies. Typical glomerular active lesions of LN were evaluated: mesangial expansion, endocapillary proliferation, glomerular deposits, extracapillary proliferation, and interstitial infiltrates, as well as tubulointerstitial chronic lesions, tubular atrophy, and interstitial fibrosis. Lesions were graded semiquantitatively using a scoring system from 0 to 3 (0 = no changes, 1 = mild, 2 = moderate, and 3 = severe). Finally, a total histological score was derived from the sum of all the described items.

Paraffin-embedded tissue sections were also stained for CD3 (Abcam, Cambridge, UK). The sections were blocked and labeled with immunoperoxidase using a VECTASTAIN ABC kit and an avidin-biotin blocking kit (Vector Laboratories, Burlingame, CA, USA) according to the manufacturer’s protocol. Peroxidase-conjugated antibody staining was followed by diaminobenzidine substrate development (Sigma-Aldrich, Madrid, Spain).

### Renal immunofluorescence studies

Slices of kidney were fixed in 4 % paraformaldehyde, embedded in Tissue-Tek® O.C.T. compound (Sakura Finetek, Alphen aan Den Rijn, the Netherlands), and stored at −80 °C. Fluorescent staining of 5-μm cryostat sections was used for confocal microscopy to quantify glomerular immunoglobulin G (IgG) and C3 deposition. Sections were directly stained with fluorescein isothiocyanate (FITC)-conjugated goat antimouse IgG (Sigma-Aldrich) and FITC-conjugated C3 (Nordic-MUbio, Susteren, the Netherlands). For analysis of C3 and IgG deposition, at least ten glomeruli were visualized and photographed with an immunofluorescence confocal microscope (Leica TCS SL spectral confocal microscope; Leica Microsystems, Mannheim, Germany). Fluorescence was quantified and normalized with Simulator-Leica confocal software (Leica Microsystems) and expressed as mean fluorescence intensity.

To quantify kidney macrophages, F4/80 immunohistochemistry was used. Briefly, 3-μm-thick kidney tissue sections embedded in paraffin were incubated with primary antibody antimouse F4/80 (eBioscience, San Diego, CA, USA) and incubated overnight at 4 °C. Staining was visualized using a secondary Alexa Fluor 546 dye (Molecular Probes/Thermo Fisher Scientific, Eugene, OR, USA). Nuclei were stained blue with DRAQ5 (eBioscience). To determine macrophage infiltration, at least 15 high-power fields were counted, and the number of positive cells was determined and expressed as a mean value.

### Statistical analysis

Overall survival was analyzed with the Kaplan-Meier method. One-way analysis of variance with post hoc tests were performed to compare proteinuria and anti-dsDNA antibodies throughout the follow-up, and gene expression and circulating cytokines were evaluated at the time the mice were killed. To compare histological data, a nonparametric Kruskal-Wallis test was used. A *p* value <0.05 was considered significant. Data are expressed as mean ± SEM.

## Results

### JAK3 inhibition prolongs survival and ameliorates renal function

To examine the effects of a JAK3 inhibitor on advanced LN in mice, we treated 6-month-old NZB/WF1 female mice with overt renal disease with CP-690,550 and compared this treatment with MMF and CYP as standard therapies. Cumulative survival analyzed with the Kaplan-Meier method was 100 % for the CYP and MMF groups, 85 % for the CP-690,550 group, and 76 % for the control group at the end of the follow-up.

**Fig. 1:**
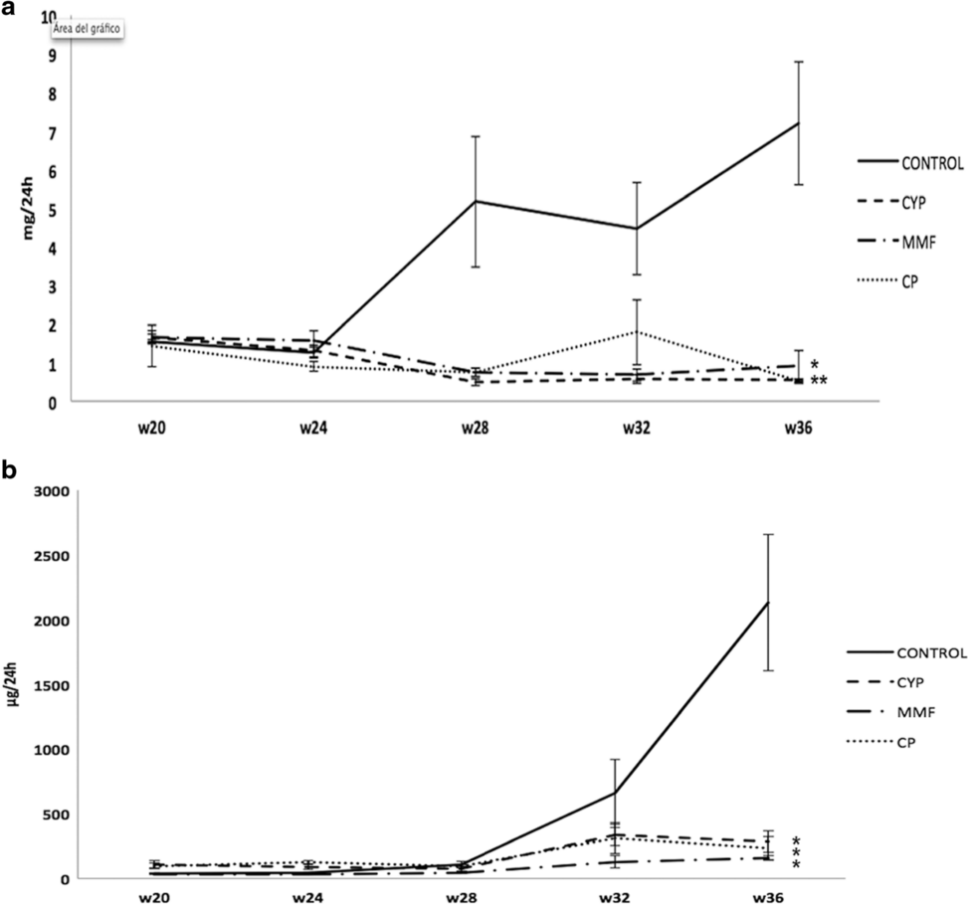
**a** Proteinuria and **b** albuminuria. Twenty-four-hour proteinuria increased progressively in PBS-treated mice (*n* = 14) to levels of heavy proteinuria. Treatment with cyclophosphamide (CYP; *n* = 14), mycophenolate mofetil (MMF; *n* = 8), and CP-690,550 (CP; *n* = 8) induced a progressive reduction of proteinuria almost to physiological levels. *p < 0.05 vs control

NZB/WF1 mice at 5 months old had slight to moderate proteinuria and albuminuria, which indicates LN. As the mice became older, without treatment they typically had severe disease, as evidenced by a progressive increase in proteinuria and albuminuria levels (Fig. 1). Twelve weeks of CP-690,550, MMF, and CYP administration resulted in significant reduction of urinary protein and albumin excretion compared with control animals. At week 36, those parameters were significantly decreased in all treated animals. Additionally, the results showed that CP-690,550-treated mice had levels of proteinuria and albuminuria similar to those of mice that received standard therapies.

### JAK3 inhibition affects the production of anti-dsDNA autoantibodies

**Fig. 2:**
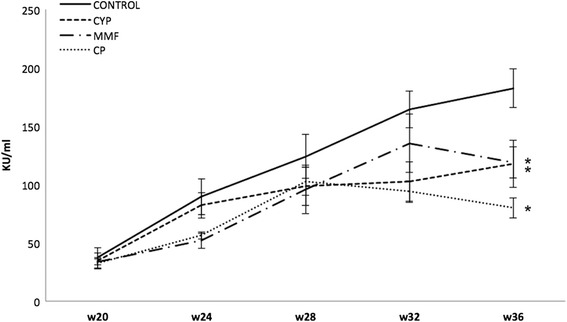
Anti-double-stranded (anti-dsDNA) antibodies. Anti-dsDNA antibodies increased progressively in PBS-treated control mice. CP-690,550 (CP) treatment reduced this production more effectively than cyclophosphamide (CYP) or mycophenolate mofetil (MMF) treatment. *p < 0.05 vs control

Serum levels of anti-dsDNA autoantibodies were determined at several time points after the beginning of treatment. As shown in Fig. 2, by age 28 weeks, serum titers of anti-dsDNA autoantibodies progressively increased in nearly all NZB/WF1 mice. In PBS-treated control animals, the results showed a severe increase of anti-dsDNA autoantibodies with disease progression.

All treatment regimens appeared to have some inhibitory effect on autoantibody production. There was a trend toward anti-dsDNA antibody reduction after week 32. Anti-dsDNA titers in the CYP-, MMF-, and CP-690,550-treated animals were significantly less than those in control mice at week 36 (*p* < 0.05). Note that while CYP broke the production of anti-dsDNA antibodies from the beginning of treatment, CP-690,550 treatment had a more profound effect and was more effective at the end of the treatment.

### JAK3 inhibition prevents glomerular and tubular lesions

**Fig. 3:**
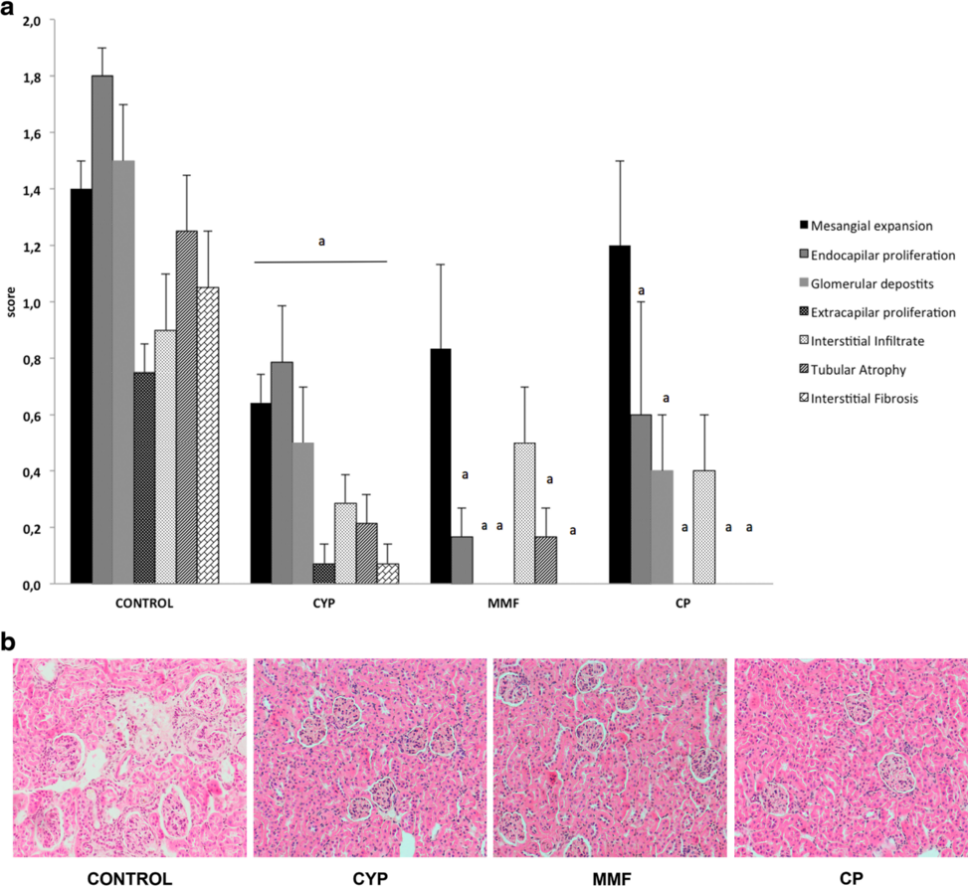
Renal histopathology. **a** Cyclophosphamide (CYP), mycophenolate mofetil (MMF), and CP-690,550 (CP) treatment reduced the elementary histological lesions of lupus nephritis (LN). **b** Representative photomicrograph of renal histology for each group (×200 original magnification, hematoxylin and eosin stain). Data are expressed as mean ± SEM. ^a^ *p* < 0.05 vs control

Renal histological analysis was performed on all surviving animals at the end of the study (Fig. 3). Typical LN lesions were seen in control mice that developed significant glomerulonephritis and interstitial inflammation. Regarding treatments, the CYP group showed a reduction in all evaluated lesions compared with vehicle-treated mice, exhibiting a pronounced reduction in endocapillary proliferation, glomerular deposits, and tubular atrophy. In the MMF group, glomerular deposits, extracapillary proliferation, and interstitial fibrosis were absent. Nevertheless, MMF animals showed important mesangial expansion and interstitial infiltrate compared with vehicle animals. CP-690,550 treatment induced a drastic reduction in endocapillary proliferation, glomerular deposits, and interstitial infiltrate, while other lesions, such as extracapillary proliferation, tubular atrophy, and interstitial fibrosis, were completely absent. Mean histological scores were as follows: CYP = 2.6 ± 0.8, MMF = 1.6 ± 0.5, and CP = 2.6 ± 1.2. These scores were statistically different with respect to the control group (control = 8.6 ± 1.1; *p* < 0.001).

### Complement and IgG glomerular deposits are reduced by JAK3 inhibition

**Fig. 4:**
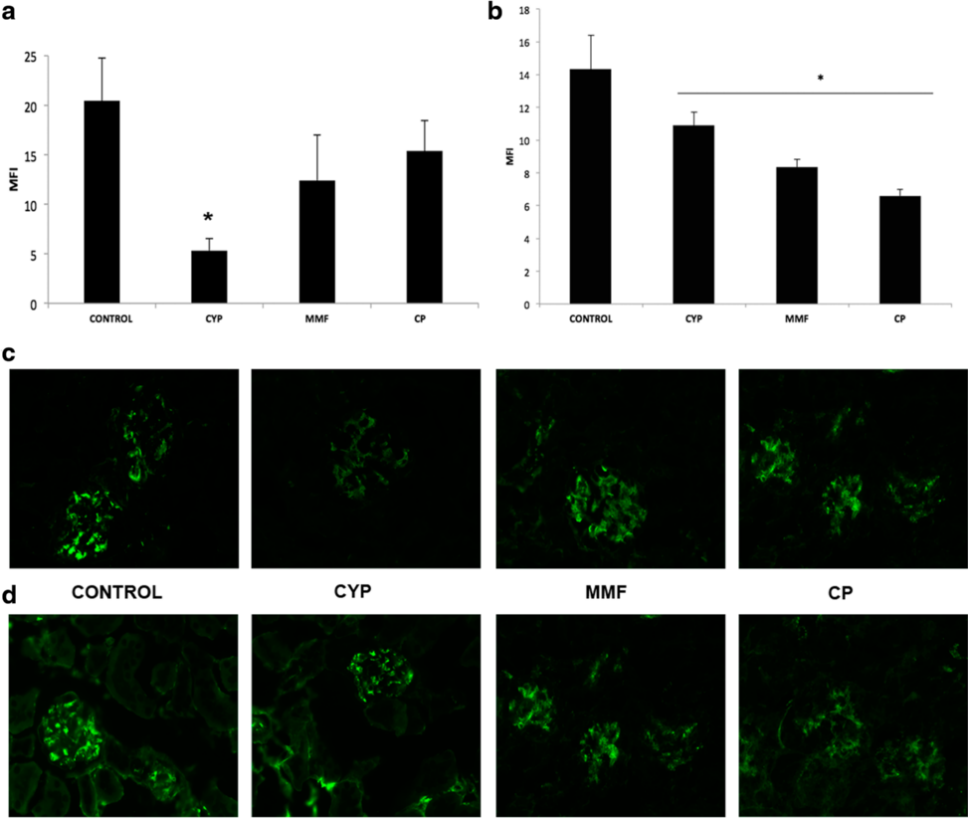
Immunofluorescence analysis of renal immunoglobulin G (IgG) and complement component 3 (C3) deposits. Deposits of renal C3 (**a**) and IgG (**b**) were quantified with confocal microscopy, and the results were expressed as mean fluorescence intensity (MFI). All treatments reduced glomerular deposits. **c** Representative photomicrographs of C3 deposits (×630 original magnification) for each group. **d** Representative photomicrographs of IgG deposits (×630 original magnification) for each group. Data are expressed as mean ± SEM. *p < 0.05 vs control. *CP* CP-690,550; *CYP* cyclophosphamide; *MMF* mycophenolate mofetil

Intrarenal deposits of IgG and complement in the glomeruli were significantly diminished in all treated animals compared with the control group (Fig. 4). IgG glomerular deposits were lower in the CP group than in the control and CYP groups. C3 glomerular deposits were also reduced in all treatment groups, although in this case CYP treatment was more effective than MMP and CP-690,550 treatment.

### Macrophages and T-cell infiltration in the glomeruli are hampered by JAK3 inhibition

**Fig. 5:**
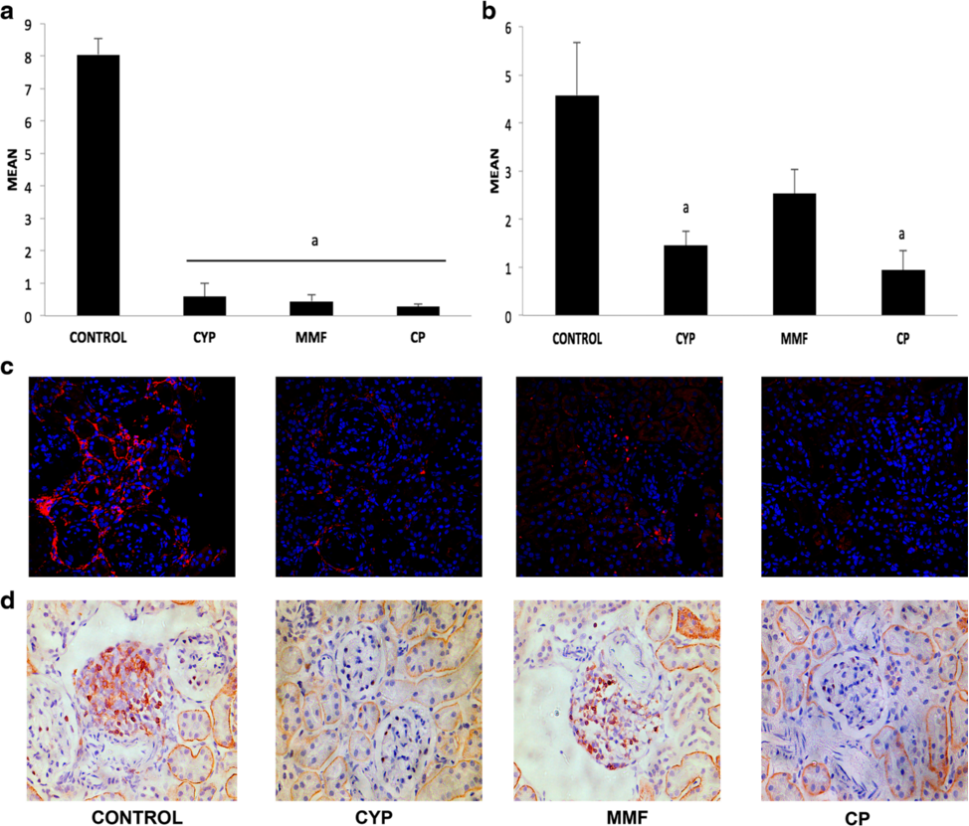
Macrophages and T-cell kidney infiltrate. **a** Kidney macrophages and **b** T-cell infiltration were quantified. All treatment clearly reduced renal infiltration. Representative photomicrographs (×200 original magnification) of (**c**) macrophage and (**d**) T-cell infiltration for each group. Data are expressed as mean ± SEM. ^a^ *p* < 0.05 vs control. *CP* CP-690,550; *CYP* cyclophosphamide; *MMF* mycophenolate mofetil

To better characterize the inflammatory cell infiltration observed with conventional histology, F4/80 (macrophage) and CD3 (T-cell) surface markers were analyzed (Fig. 5). The number of cells that stained positive for F4/80 was significantly reduced in all treated groups compared with the control group. As can be seen in the photomicrographs in Fig. 5, macrophages were localized mainly around the glomeruli close to the Bowman’s capsules and in the interstitial area.

T-lymphocyte infiltration evidenced by CD3 immunostaining showed that, again, all treatments affected kidney CD3 infiltration. Infiltrating CD3^+^ cells were localized in the tubule-interstitium space, where dendritic cells and other inflammatory cells are commonly found.

### JAK3 inhibition modulates renal inflammatory gene expression

Expression analysis of different inflammatory genes in the kidney (Table 1) revealed that there was a significant upregulation in their expression compared with healthy animals. The JAK-STAT pathway is key in the pathogenesis of LN, so vehicle-treated control animals displayed an elevated expression of STAT genes that signal downstream in the cascade of JAK receptors (STAT1, STAT2, STAT3, STAT4, and STAT5a). All treatments induced a reduction in expression of these genes, achieving almost healthy levels, which indicates good inhibition of the pathway by all the compounds, especially CP-690,550. C3 gene expression, as a manifestation of local complement synthesis, was significantly reduced in all treatments. These results paralleled the C3 glomerular deposits. CCL2 and CCL5 are genes related to the recruitment of several inflammatory cells into inflammatory sites, and they play an important role in the recruitment of monocytes, T cells, dendritic cells, eosinophils, and so forth. The results showed that all of these genes were diminished in treated animals. Another gene that participates in cell adhesion is VCAM. Its expression was reduced by all treatments, according to the kidney cell infiltration results. The costimulatory pathway was also affected by CYP, MMF, and CP-690,550 treatment. CD40L was reduced in all treated animals. An intense local effect in inflammatory cytokine expression was observed. IL-2 and IL-6 were significantly reduced in all treatment groups. There was no evidence of activation of TLR4 (data not shown). TLR9, a sensor of dsDNA, was overexpressed in vehicle-treated animals and was strongly reduced by all treatments.

**Table 1:** Kidney gene expression

Gene	Control	CYP	MMF	CP	*p* Value
*C3*	4.8 ± 1.6	1.61 ± 0.4^a^	1.67 ± 0.2^a^	2.1 ± 0.4^a^	0.02
*CCL2*	3.5 ± 0.9	1.35 ± 0.4	2.7 ± 0.9	2 ± 0.6	NS
*CCL5*	1.4 ± 0.4	0.43 ± 0.1	0.9 ± 0.2	0.9 ± 0.4	NS
*CD40L*	1.2 ± 0.3	0.4 ± 0.1^a,b^	0.8 ± 0.2	0.1 ± 0.1^a,b^	0.0017
*IL2*	1.3 ± 0.4	0.5 ± 0.2^a^	0.3 ± 0.1^a^	0.2 ± 0.1^a^	0.02
*IL6*	3.3 ± 1.7	0.3 ± 0.0^a^	0.9 ± 0.3^a^	0.5 ± 0.1^a^	0.03
*TLR9*	1.5 ± 0.1	0.3 ± 0.1^a^	0.5 ± 0.1^a^	0.6 ± 0.1^a^	0.0001
*VCAM1*	1.2 ± 0.1	0.8 ± 0.2	0.6 ± 0.1	0.9 ± 0.1	NS
*STAT1*	2.01 ± 0.2	0.66 ± 0.3^a^	0.71 ± 0.1^a^	0.48 ± 0.1^a^	0.0001
*STAT2*	1.86 ± 0.1	0.82 ± 0.1^a^	0.86 ± 0.1^a^	0.57 ± 0.1^a^	0.0001
*STAT3*	2.14 ± 0.4	0.92 ± 0.1^a^	0.99 ± 0.2	0.98 ± 0.1^a^	0.016
*STAT4*	2.23 ± 0.3	0.42 ± 0.1^a^	0.51 ± 0.1^a^	0.21 ± 0.1^a^	0.0001
*STAT5a*	1.42 ± 0.3	0.64 ± 0.1^a^	0.59 ± 0.1^a^	0.62 ± 0.1^a^	0.021

*Abbreviations: C3*, complement component 3, *CCL* chemokine (C-C motif) ligand, *CP* CP-690,550, *CYP* cyclophosphamide, *IL* interleukin, *MMF* mycophenolate mofetil, *NS* not significant, *STAT* signal transducer and activator of transcription, *TLR* Toll-like receptor, *VCAM1* vascular cell adhesion molecule 1

Data presented are fold changes in 18S ribosomal RNA, house keeping gene for random target polymerase chain reaction

^a^Compared with control

^b^Compared with MMF

### JAK3 inhibition decreases proinflammatory systemic circulating cytokines

**Fig. 6:**
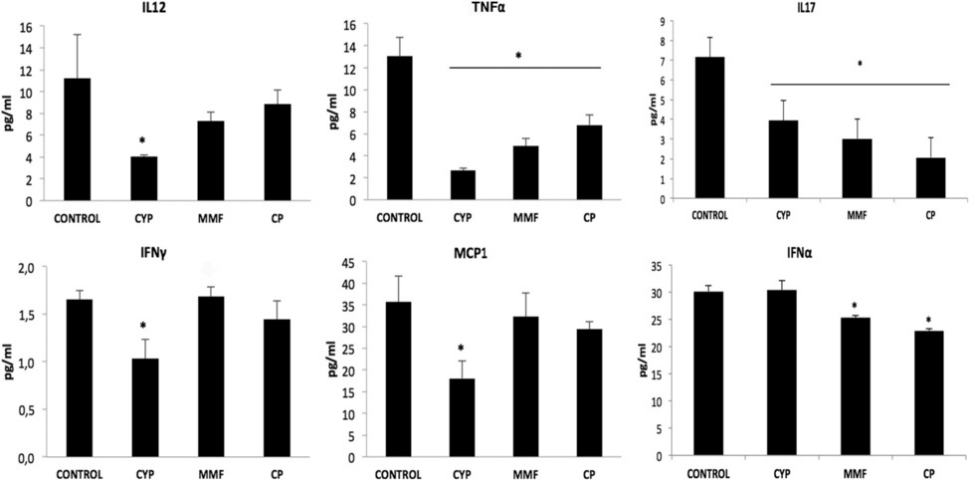
Systemic circulating inflammatory cytokines. Lupus nephritis promoted overexpression of proinflammatory cytokines in serum. Treatment with cyclophosphamide (CYP), mycophenolate mofetil (MMF), and CP-690,550 (CP) reduced their levels. Data are expressed as mean ± SEM. *p < 0.05 vs control. *IFN* interferon, *IL* interleukin, *MCP* monocyte chemoattractant protein, *TNF* tumor necrosis factor

To determine whether the different treatments modified the systemic inflammatory response, we measured serum levels of some proinflammatory cytokines (Fig. 6). LN induced an increase in the secretion of different pathogenic proinflammatory cytokines, such as IL-12, IL-17, TNF-α, MCP-1, IFN-α, and IFN-γ, compared with healthy animals (data not shown). CYP treatment induced a reduction in almost all these cytokines. Of note, CYP treatment did not affect IFN-α circulating levels. MMF treatment appeared to be less immunomodulatory, considering secretion of this cytokine. JAK3 inhibition showed a trend toward reducing all the evaluated Th1 cytokines, but it is noteworthy that there were significant reductions in the secretion of TNF-α, IFN-α, and IL-17.

## Discussion

With recent insight into the pathological role of JAK-STAT proteins in the inflammatory process, it has become feasible to target this pathway in order to modulate their pathogenic effects. In this study, we have demonstrated that treatment of NZB/WF1 mice, a well-recognized experimental murine model of autoimmune nephritis, with the JAK3 inhibitor CP-690,550 resulted in improved animal survival, delayed the progression of the disease, and ameliorated renal function, preventing kidney cell infiltration and complement deposition. We have also proven that treatment with CP-690,550 reduced the severity of typical tubular and glomerular lesions and that JAK-STAT inhibition had an impact on autoantibody formation, as illustrated by a reduction in circulating anti-dsDNA antibody titers, a hallmark of the disease. As expected, either renal mRNA expression or circulating inflammatory cytokine signaling through the JAK-STAT pathway was diminished.

CYP has been used as a standard treatment for SLE and LN, but its toxicity and relatively low effectiveness in preventing disease relapse have made its use less common; more than 30 % of patients with LN relapse within 3 years after cessation of therapy. In addition to CYP, we included MMF in our experimental design, which nowadays is becoming a reference drug for the treatment of patients with LN [21]. In our present study, we have proven in an experimental mouse model that both drugs exert similar potent effects in all the evaluated parameters, as previously reported in patients [21].

The role played by proinflammatory cytokines in the pathogenesis of LN has been clearly established [22, 23]. One of the promising developments in the last decade has been the identification of the function of the IL-23/Th17 pathway in autoimmune disease initiation, progression, and maintenance. Recent studies have demonstrated that either circulating IL-17 or Th17 cells by themselves were positively correlated with the Systemic Lupus Erythematosus Disease Activity Index [24]. A strong correlation was observed in serum levels of IL-17 in patients with SLE, suggesting that IL-17 may drive activation of diverse autoimmune pathways. Many of the cytokines involved in LN and other autoimmune diseases, including IL-17, signal through receptors associated with JAKs, and CP-690,550 targets JAK3, which plays a pivotal role in the beginning of the inflammatory cytokine signaling pathway. In this sense, the present study demonstrates that inhibition of the JAK-STAT pathway is essential in preventing proinflammatory cytokines from fulfilling their role.

LN is characterized by the presence of circulating autoantibodies as well as deposition of immune complexes or components in renal tissue [25, 26]. As our results show, CP-690,550 treatment significantly reduced the deposition of IgG and C3 in the glomeruli, probably as a result of the inhibition of circulating autoantibodies, as reflected by the reduced anti-dsDNA serum levels in treated animals. Also, our results suggest that CP-690,550 is more effective than CYP or MMF in the inhibition of anti-dsDNA autoantibody formation. In other studies, researchers have demonstrated the influence of CP-690,550 in autoantibody formation. Yamamoto et al. [27] showed that disease activity in patients with rheumatoid arthritis with insufficient response to methotrexate and patients with SLE decreased with CP-690,550 treatment, and that the elevated titers of both rheumatoid factor and anti-DNA antibodies became negative.

A crucial pathological feature of LN is the inflammatory infiltration triggered by tissue immune deposition [28, 29]. Our results show a prominent infiltration of T cells and macrophages in renal tissue in vehicle-treated animals. Also, there was an increase in serum MCP-1, as well as prominent renal gene expression of regulated on activation, normal T cell expressed and secreted (RANTES) and MCP-1, which are potent chemoattractants for monocytes and T cells [30]. CP-690,550 treatment significantly reduced both T-cell and macrophage kidney infiltration, with a slight reduction in serum MCP-1 but a greater reduction in MCP-1 and RANTES renal gene expression. Finally, the renal reduction of all STAT components, indicating local inhibition of the downstream JAK-STAT pathway, may participate in the modulation of the renal inflammatory microenvironment.

Some other authors have demonstrated that treatment of lupus-prone mice with JAK2 inhibitors led to disease prevention or improvement. Wang et al. showed that JAK2 inhibition in MRL/lpr mice led to a decrease in proteinuria, serum levels of dsDNA, renal cell infiltration, and deposition of IgG and C3 in the kidney [31]. Lu et al. used another JAK2 inhibitor in NZB/WF1 mice and showed improved survival, reduced proteinuria, and diminished dsDNA antibodies and spleen plasma cells; additionally, several cytokines were downregulated with treatment [32]. However, all of these studies were focused on the inhibition of JAK2; JAK2 specifically mediates cytokine signaling for red blood cells and platelets, and its inhibition causes anemia and low platelets [33, 34]. The results of our present study clearly show that selective inhibition of JAK3 is ready to use as a new treatment for LN in clinical practice, given its profile and good tolerability in autoimmune diseases.

Apart from conventional inflammatory cytokines, gene expression analysis revealed the reduction of other mediators in renal tissue. TLR9 was, as expected, increased in control animals, and it was significantly reduced by all treatments. We also observed that the JAK-STAT pathway affected CD40L expression; blocking this pathway induced a severe and significant reduction of its expression, thereby affecting CD40-CD40L interaction. It is well known that this dyad plays a central role in the development of immune-mediated inflammatory disease processes [35, 36], and we previously showed that blocking this pathway was also effective in the prevention of LN [37].

Finally, in our present study, we observed a dramatic drop in circulating levels of IL-12, IFN-α, and TNF-α in animals that received CP-690,550 treatment. The influence of JAK3 inhibition on inflammatory cytokines was also observed by Ghoreschi et al. [20], who reported a rapid improvement of the disease with CP-690,550 in a model of established arthritis, with inhibition of inflammatory mediators such as IFN-γ, IL-6, IL-12, and IL-23 in joint tissue. Further, Rosengren et al. showed that CP-690,550 decreased TNF-induced chemokine expression in fibroblasts from patients with rheumatoid arthritis [38]. No previous information has been reported regarding the effects of JAK inhibitors on IL-17 in experimental LN, but in our present study CP-690,550 treatment resulted in a consistent reduction of circulating IL-17 levels. In fact, the STAT3 transcription factor is essential for the differentiation of Th17 cells [7], and it is effectively inhibited by CP-690,550. As circulating IL-17A has been associated with immune complex deposition and complement activation in kidneys in a mouse model of lupus [8], it is quite likely that reduction of IL-17 in the bloodstream has a systemic mechanism that participates in the reduction of renal inflammation.

## Conclusions

Our data strongly support the idea that the JAK-STAT pathway is implicated in the progression of renal inflammation in NZB/WF1 mice. Our results also provide evidence of the potential therapeutic effects of targeting this pathway with CP-690,550 as part of a robust strategy of immune disruption in autoimmune LN disease, particularly when treatment is initiated during the early phases of the disease. This has the potential to become a powerful new therapeutic tool in the treatment of LN and other autoimmune diseases.

## Abbreviations

ATP, adenosine triphosphate; C3, complement component 3; CBA, cytometric bead array; CCL, chemokine (C-C motif) ligand; CP, CP-690,550; CYP, cyclophosphamide; dsDNA, double-stranded DNA; ELISA, enzyme-linked immunosorbent assay; FITC, fluorescein isothiocyanate; IC, immune complex; IFN, interferon; IgG, immunoglobulin G; IL, interleukin; JAK, Janus kinase; LN, lupus nephritis; MCP-1, monocyte chemoattractant protein 1; MFI, mean fluorescence intensity; MMF, mycophenolate mofetil; PCR, polymerase chain reaction; RANTES, regulated on activation, normal T cell expressed and secreted; SLE, systemic lupus erythematosus; STAT, signal transducer and activator of transcription; TLR, Toll-like receptor; TNF, tumor necrosis factor; VCAM-1, vascular cell adhesion molecule 1
