# Erratum to: JAK3-STAT pathway blocking benefits in experimental lupus nephritis

**DOI:** 10.1186/s13075-016-1058-2

**Published:** 2016-07-01

**Authors:** Èlia Ripoll, Laura de Ramon, Juliana Draibe, Ana Merino, Nuria Bolaños, Montse Goma, Josep M. Cruzado, Josep M. Grinyó, Juan Torras

**Affiliations:** Laboratori 4120. Nefrologia Experimental, 4a Planta Pavelló Govern, Universitat de Barcelona. Campus Bellvitge, Institut d’Investigació Biomèdica de Bellvitge (IDIBELL). Departament de Nefrologia, Hospital Universitari de Bellvitge, E-08907 L’Hospitalet, Barcelona, Spain; Departament d’Anatomia Patològica, Hospital Universitari de Bellvitge, E-08907 L’Hospitalet, Barcelona, Spain

Unfortunately, after publication of this article [1], it was noticed that the name of Juliana Draibe was listed incorrectly. The corrected name can be seen in the author list above and the original article has been updated to reflect this change.
